# Supplementary material for: Chemotaxis-driven delivery of nano-pathogenoids for complete eradication of tumors post-phototherapy
Source: Nat Commun. 2020 Feb 28;11:1126. doi: 10.1038/s41467-020-14963-0 (PMC7048836; doi:10.1038/s41467-020-14963-0)
Supplement: Supplementary file 1 — Supplementary Information [file 41467_2020_14963_MOESM1_ESM.pdf]

Supplementary Information

Chemotaxis-Driven Delivery of Nano-Pathogenoids for Complete Eradication of Tumors Post-Phototherapy

Min Li *et al*.

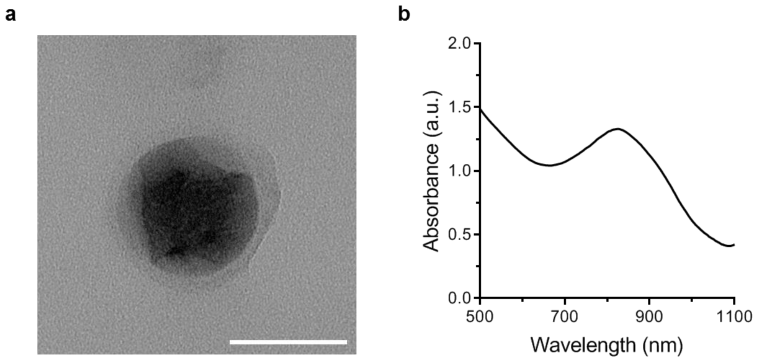

Supplementary Figure 1 Characterization of NPs@PBT. (**a**) Transmission electron microscopy (TEM) imaging and (**b**) absorbance spectrum of NPs@PBT. Scale bar, 100 nm. Data are representative of three independent samples. Source data are provided as a Source Data file.

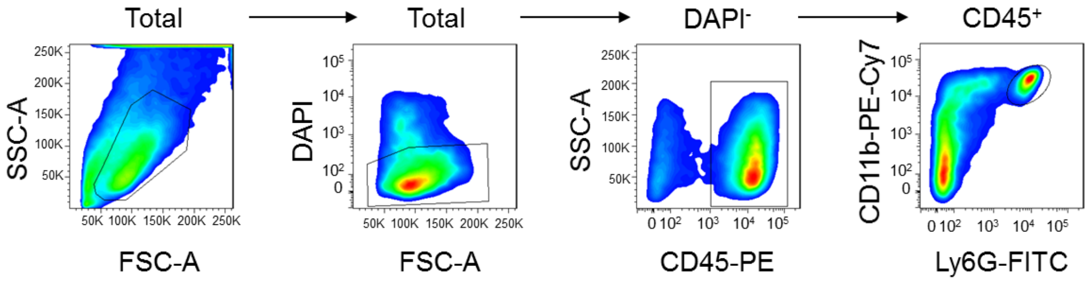

Supplementary Figure 2 Gating strategy for flow cytometric studies of neutrophils. Gating strategy to analyze the percentage of neutrophils ($CD11b^{+}Ly6G^{+}$) in $DAPI^{-}$ $CD45^{+}$ leukocytes (Fig. 2f, g, i-k and Fig. 5e, f, h, i).

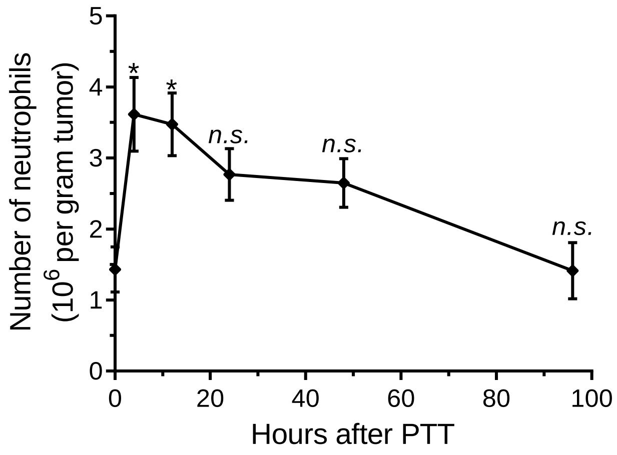

Supplementary Figure 3 Number of neutrophils per gram of tumor at indicated time points post PTT. *n* = 3 mice per time point. Data are shown as mean ± SEM and analyzed by unpaired two-tailed Student's *t*-test. *$P < 0.05$, *n.s.*, not significant. Source data are provided as a Source Data file.

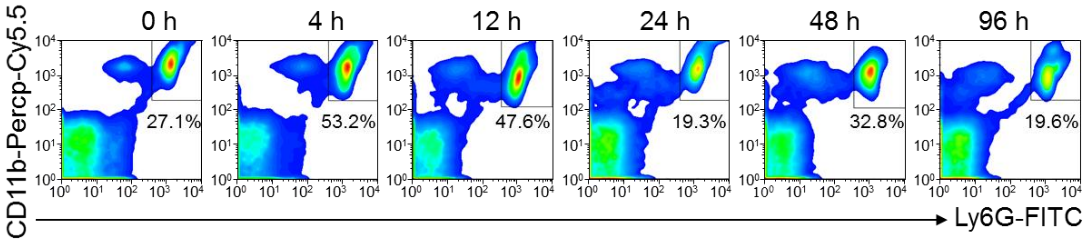

Supplementary Figure 4 PTT induced an increased percentage of neutrophils in blood. Tumor sites of EMT6-bearing mice were photothermally treated with 40 ºC for 5 min. The percentages of neutrophils ($CD11b^+Ly6G^+$) in $CD45^+$ blood leukocytes at different time points were analyzed by flow cytometry.

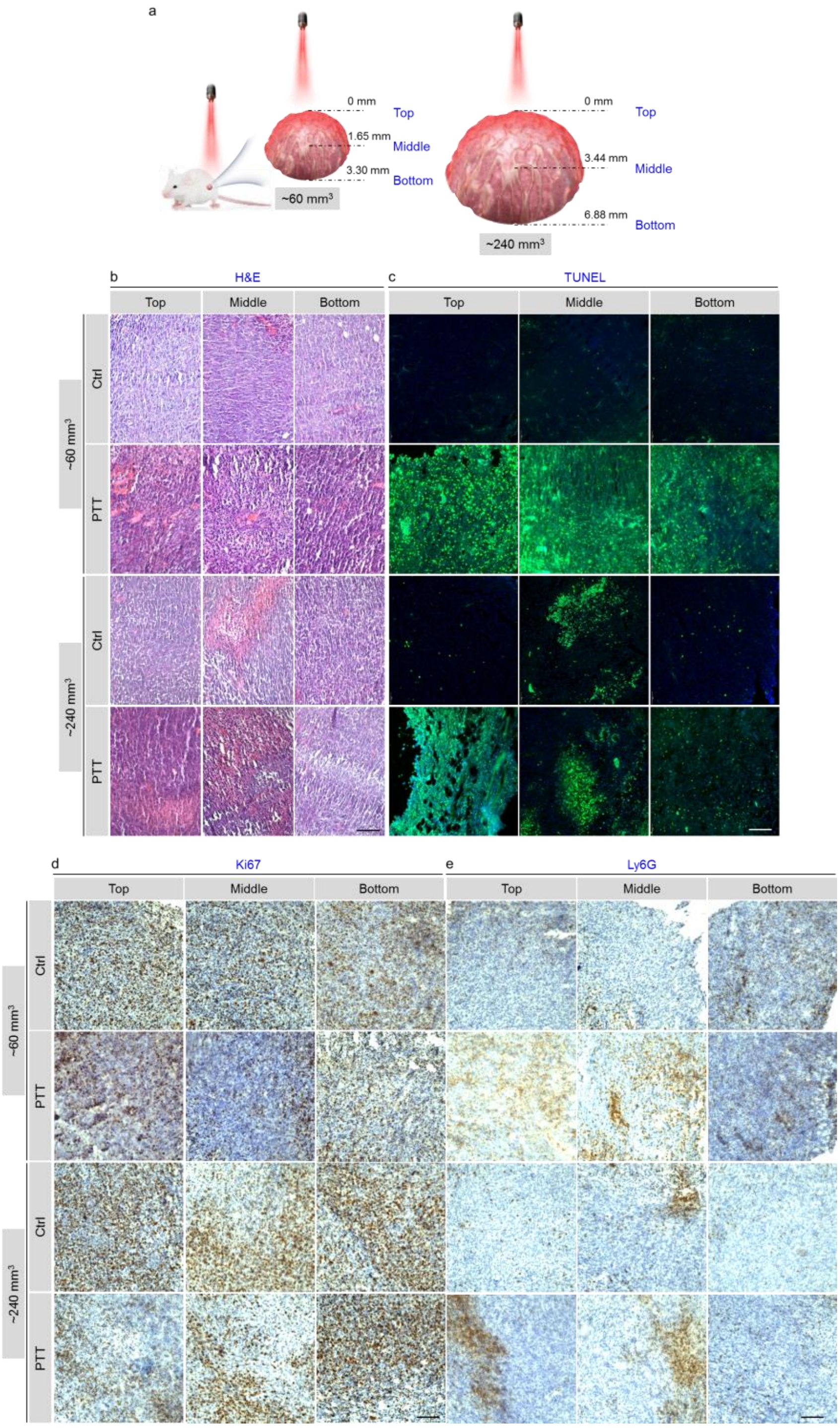

Supplementary Figure 5 PTT induced less cellular apoptosis and neutrophil recruitment in the deeper regions of larger tumors. EMT6 tumors with sizes of ~60 or 240 mm^3 were treated with PTT (40 °C for 5 min) or left untreated as a control. After 4 h, the tumors were obtained and dissected to observe the cellular apoptosis and proliferation as well as neutrophil infiltration at different depths inside the tumor tissues. **a** Schematic showing analyses of PTT effects in tumor regions at different depths under

the irradiated skin. (**b**-**e**) Representative images of (**b**) H&E, (**c**) terminal deoxynucleotidyl transferase-mediated dUTP nick end-labeling (TUNEL), (**d**) Ki67, and (**e**) Ly6G staining of tumor slices obtained at different depths inside the tumors. Scale bar, 100 μm. Data are representative of two independent experiments.

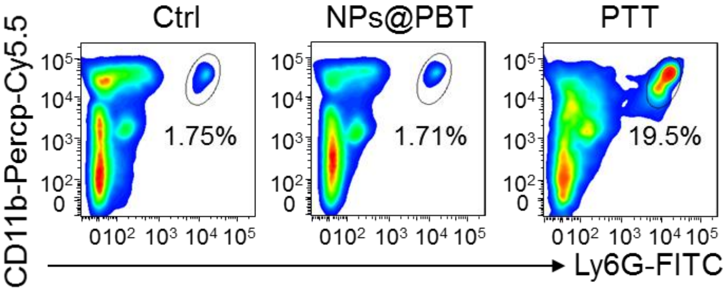

Supplementary Figure 6 PTT induced an increased percentage of neutrophils in tumors. CT26-bearing mice were left untreated, *i.v.* injected with NPs@PBT alone or photothermally treated with 40 ºC for 5 min at tumor tissue. At 4 h post treatment, the percentage of neutrophils ($CD11b^+Ly6G^+$) in $DAPI^-CD45^+$ tumor-infiltrating leukocytes was detected by flow cytometry.

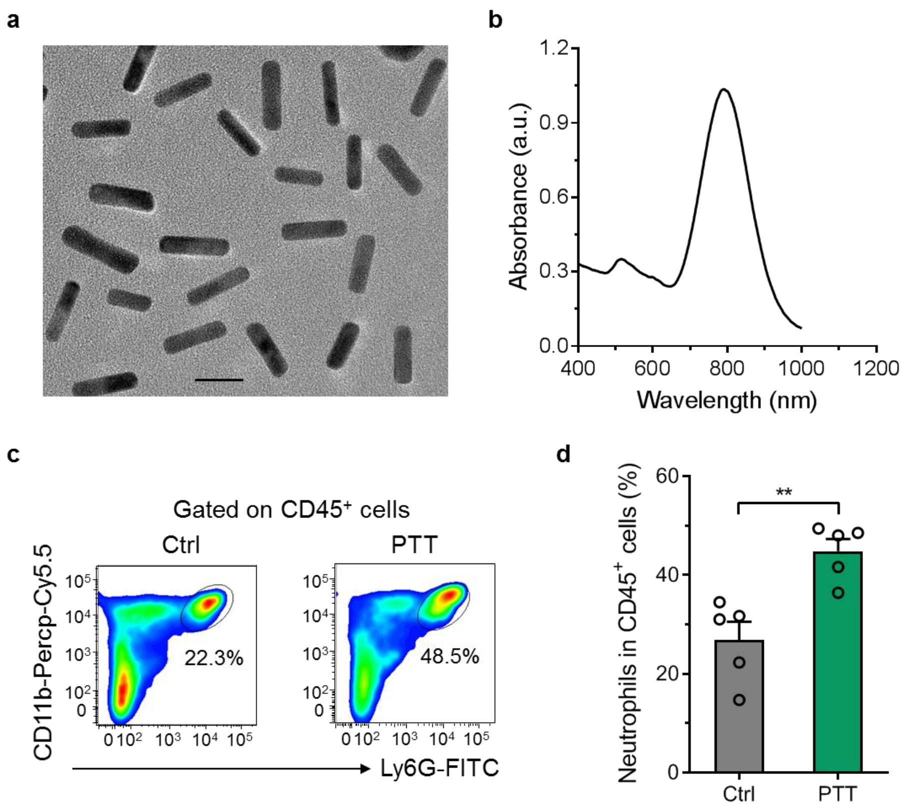

Supplementary Figure 7 PTT using gold nanorods (GNRs) as transducer induced neutrophil recruitment into tumor. (**a**) TEM imaging and (**b**) absorbance spectrum of GNRs. Data are representative of three independent experiments. **c**, **d** PTT using GNRs as transducer caused neutrophil accumulation in tumors. EMT6-bearing mice were left untreated, injected with GNRs and photothermally treated with 40 ºC for 5 min of tumor tissue. At 4 h post PTT, the percentage of neutrophils ($CD11b^{+}Ly6G^{+}$) in $DAPI^{-}CD45^{+}$ tumor-infiltrating leukocytes was detected by flow cytometry. $n = 5$ per group. Data are shown as mean ± SEM and analyzed by unpaired two-tailed Student's *t*-test. $^{**}P < 0.01$. Scale bar, 20 nm. Source data are provided as a Source Data file.

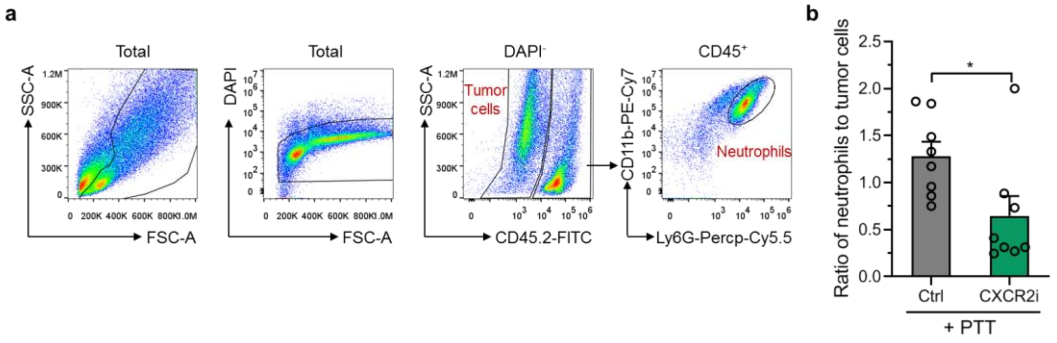

Supplementary Figure 8 Inhibition of CXCR2 decreased neutrophil recruitment to PTT-treated tumors. EMT6-bearing mice were treated with PTT on tumors 30 min post injection of CXCR2 inhibitor SB225002 (CXCR2i). Mice pre-injected with 0.33% tween-80 and 10% DMSO in PBS were used as the control. After 4 h, tumors were excised and analyzed by flow cytometry. **a** Gating strategy to analyze populations of neutrophils and tumor cells in the tumors. **b** The ratio of neutrophils to tumor cells in the tumors after the indicated treatments. $n = 8$ per group. Data are shown as mean ± SEM and analyzed by unpaired two-tailed Student's t-test. *$P < 0.05$. Source data are provided as a Source Data file.

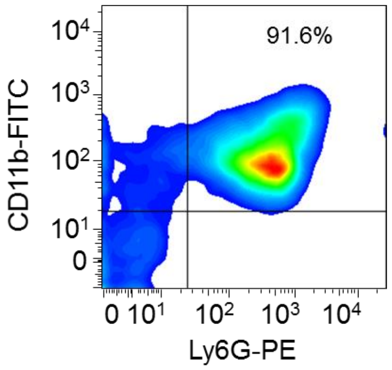

Supplementary Figure 9 The purity of neutrophils isolated from bone marrow.

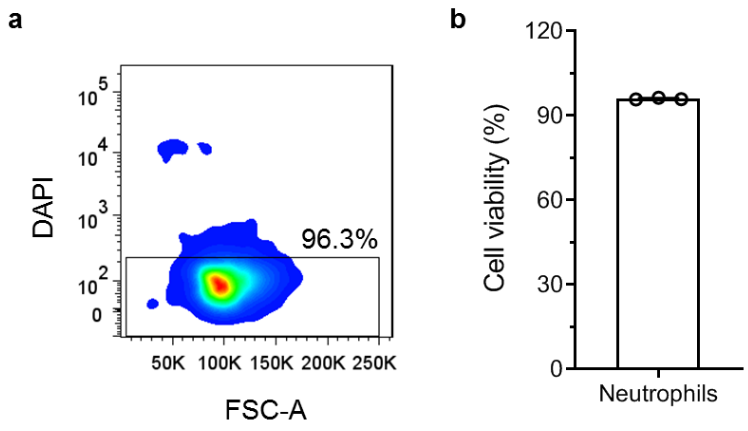

Supplementary Figure 10 The viability of isolated neutrophils. **a** Viability of isolated neutrophils was determined by DAPI staining. **b** Viability of isolated neutrophils was presented as a bar graph. *n* = 3 biologically independent samples. Data are shown as mean ± SD. Source data are provided as a Source Data file.

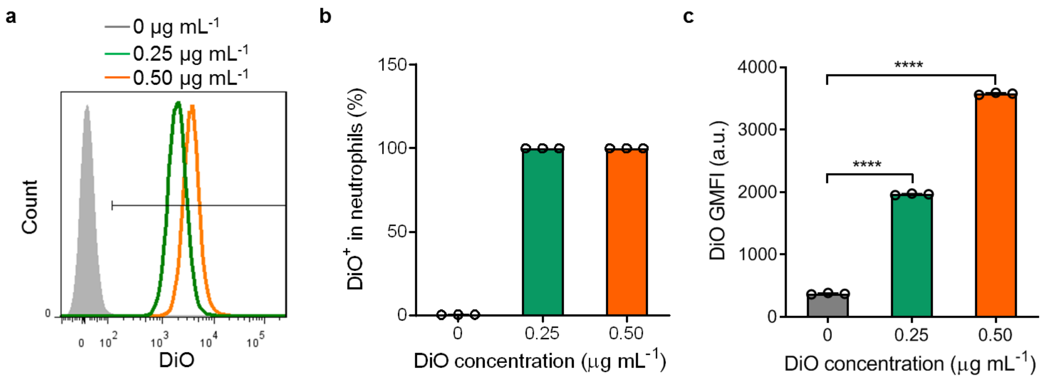

Supplementary Figure 11 Neutrophils have a strong capacity to engulf NPs. Neutrophils were cultured with NPs@DiO or not at a DiO concentration of 0, 0.25 and 0.50 μg mL^{-1} for 1 h. The percentage of neutrophils containing NPs@DiO was analyzed by flow cytometry. **a** Almost all neutrophils contained NPs@DiO. **b** The percentage of neutrophils containing NPs@DiO was presented as a bar graph. **c** DiO GMFI of neutrophils after different treatments. (**b**, **c**) $n = 3$ biologically independent samples per group. Data are shown as mean ± SD and analyzed by unpaired two-tailed Student's *t*-test. ****$P < 0.0001$. Source data are provided as a Source Data file.

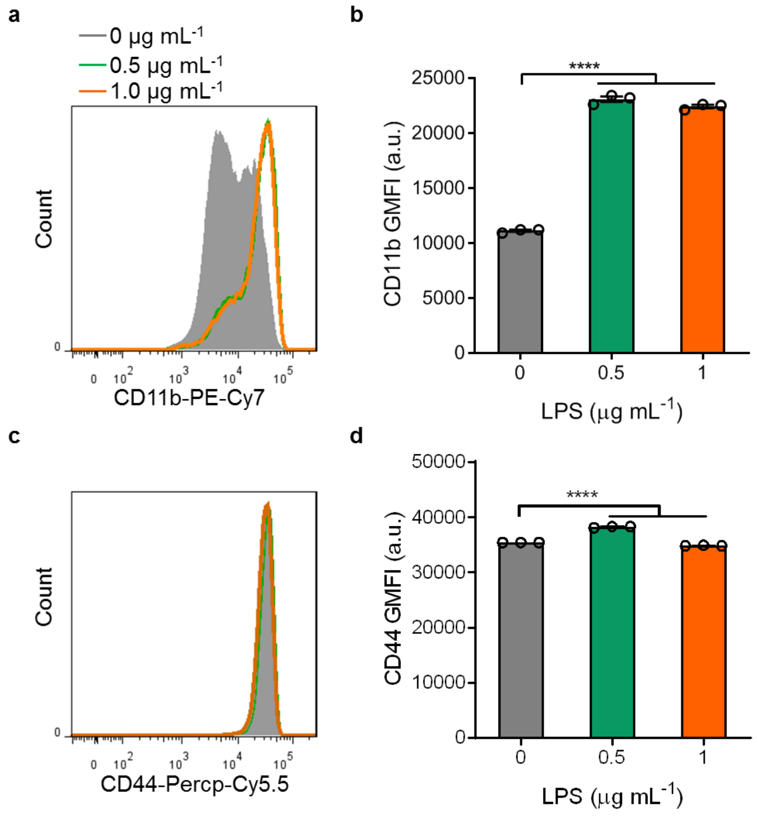

Supplementary Figure 12 Enhanced expression of CD11b and CD44 on neutrophils in response to LPS stimulation. Neutrophils were cultured with LPS or not at a concentration of 0, 0.5 and 1.0 μg mL^{-1} for 1 h. **a** CD11b expression on neutrophils was determined by flow cytometry. **b** CD11b GMFI of neutrophils after different treatments. **c** CD44 expression on neutrophils was determined by flow cytometry. **d** CD44 GMFI of neutrophils after different treatments. (**b**, **d**) n = 3 biologically independent samples per group. Data are shown as mean ± SD and analyzed by unpaired two-tailed Student's t-test. ****$P < 0.0001$. Source data are provided as a Source Data file.

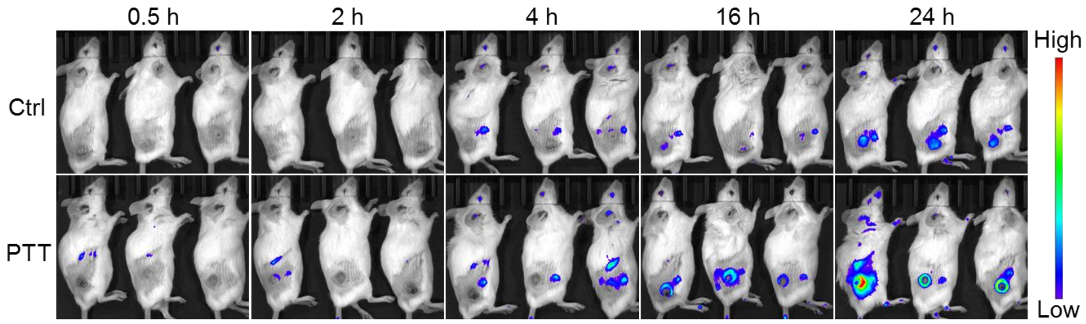

Supplementary Figure 13 PTT induced accumulation of transferred neutrophils into the tumor tissues. DiD-labelled neutrophils were *i.v.* injected into control or photothermally treated EMT6-bearing mice. At different time points, *in vivo* DiD signals were observed with *in vivo* imaging system (IVIS). *n* = 3 per group.

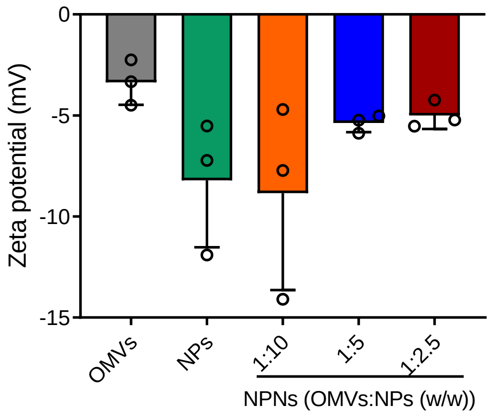

Supplementary Figure 14 Zeta potential values of OMVs, NPs, and NPNs with different proportion of OMVs to NPs. *n* = 3 biologically independent samples per group. Data are shown as mean ± SD. Source data are provided as a Source Data file.

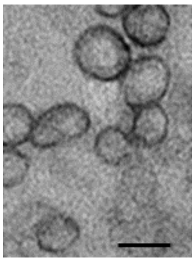

Supplementary Figure 15 A typical TEM image of NPNs, which showed the core-shell structure. Scale bar, 50 nm. Data are representative of two independent experiments.

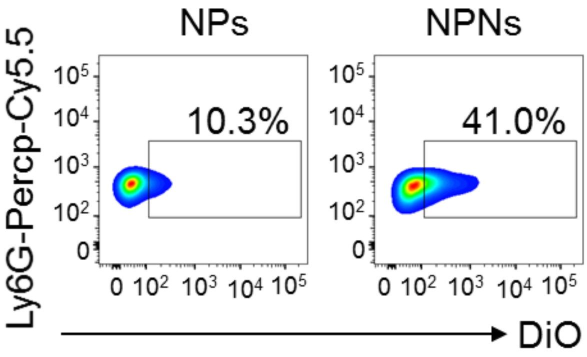

Supplementary Figure 16 Representative flow cytometric analyses of percentage of neutrophils that contained NPs or NPNs in blood.

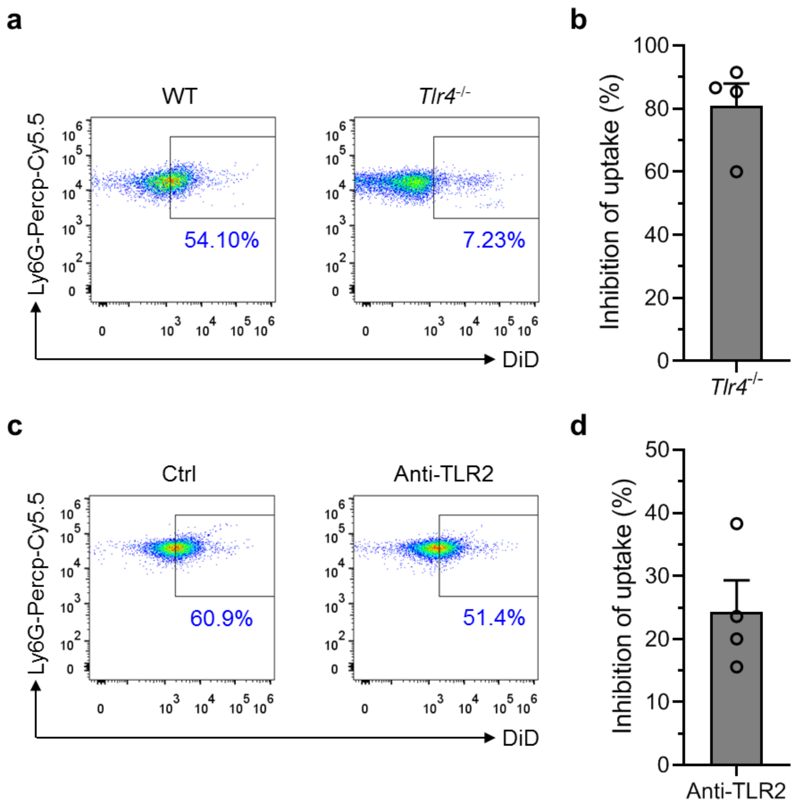

Supplementary Figure 17 Uptake of NPNs@DiD by neutrophils in TLR4 knockout mice (*Tlr4*$^{-/-}$) and mice pre-treated with anti-TLR2 antibody. **a** Flow cytometric analyses of the percentage of neutrophils that contained NPNs@DiD (defined as Ly6G^{+}DiD^{+} cells). Wild type (WT) mice or *Tlr4*$^{-/-}$ mice were injected with NPNs@DiD and subjected to flow cytometric analyses at 4 h post injection. **b** The inhibition rate of NPNs uptake by neutrophils in *Tlr4*$^{-/-}$ mice relative to WT mice. n = 4 per group. **c** Flow cytometric analyses of the percentage of neutrophils that contained NPNs@DiD (defined as Ly6G^{+}DiD^{+} cells) in mice pre-treated with anti-TLR2 antibody. The mice were injected with NPNs@DiD and subjected to flow cytometric analyses at 4 h post injection. **d** The inhibition rate of NPNs uptake by neutrophils in anti-TLR2 treated mice relative to the untreated mice. n = 4 per group. Data are shown as mean ± SEM. Source data are provided as a Source Data file.

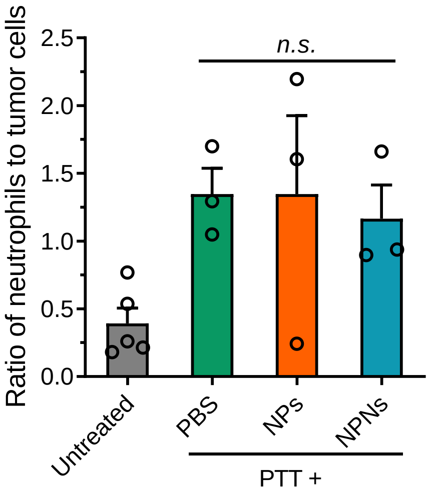

Supplementary Figure 18 Uptake of NPNs by neutrophils did not alter neutrophil infiltration into tumors. EMT6 bearing mice were treated with PTT and then *i.v.* injected with PBS, PEG-*b*-PLGA NPs, or NPNs. After 4 h, the ratio of neutrophils to tumor cells in the tumors was analyzed by flow cytometry. n = 5 for untreated group and n = 3 for the other three groups. Data are shown as mean ± SEM and analyzed by unpaired two-tailed Student's *t*-test. *n.s.*, not significant. Source data are provided as a Source Data file.

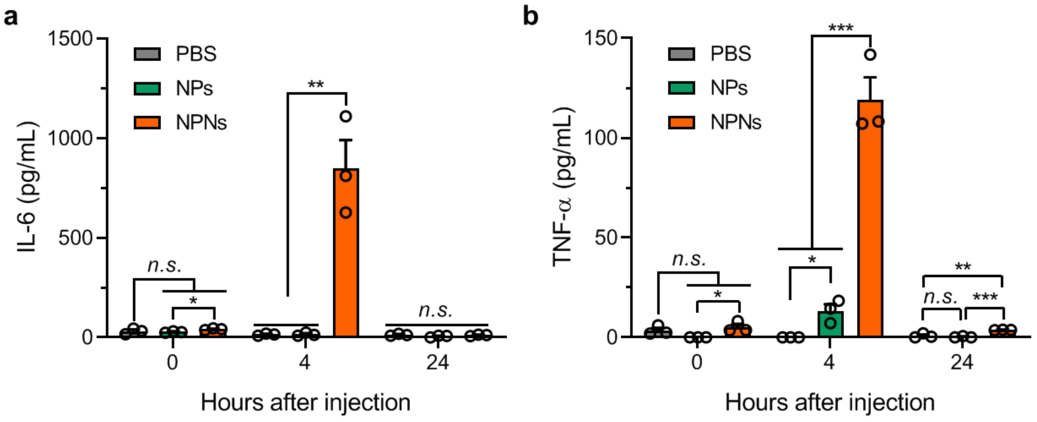

Supplementary Figure 19 Injection of NPNs induced a transient increase of proinflammatory cytokines. Concentrations of pro-inflammatory cytokines (**a**) IL-6 and (**b**) TNF-α in serum at the indicated time points post injection of PBS, NPs, and NPNs were detected by ELISA. Data are shown as mean ± SEM and analyzed by unpaired two-tailed Student's *t*-test. $n = 3$ per group. *$P < 0.05$, **$P < 0.01$, ***$P < 0.001$. *n.s.*, not significant. Source data are provided as a Source Data file.

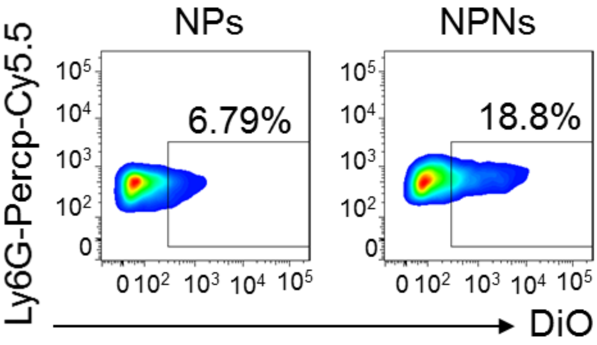

Supplementary Figure 20 Representative flow cytometric images of neutrophils encapsulating NPs and NPNs in tumor.

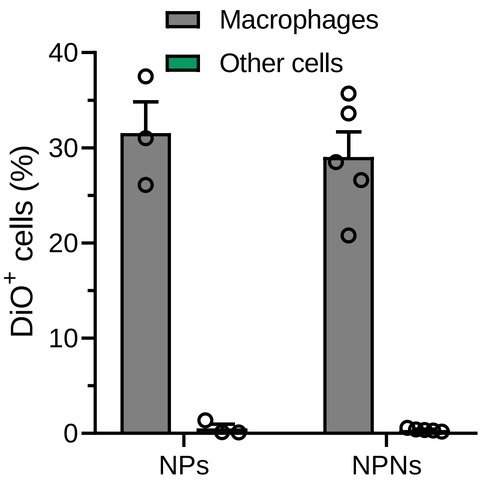

Supplementary Figure 21 Uptake of NPs@DiO and NPNs@DiO by macrophages and other non-neutrophil immune cells in PTT-treated tumors. DiO-labelled NPs and NPNs were *i.v.* injected into PTT-treated EMT6-bearing mice. At 4 h post injection the percentage of macrophages and other non-neutrophil immune cells encapsulating NPs and NPNs was analyzed by flow cytometry. n = 3 for NPs group and n = 5 for NPNs group. Data are shown as mean ± SEM. Source data are provided as a Source Data file.

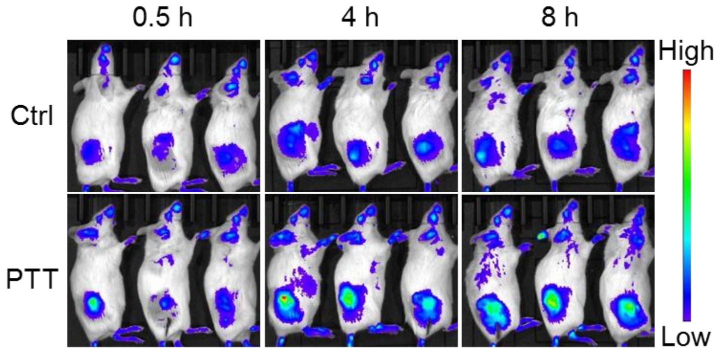

Supplementary Figure 22 More NPNs accumulated in PTT-treated tumors. DiD-labelled NPNs were *i.v.* injected into control or photothermally treated EMT6-bearing mice. At different time points, *in vivo* DiD signals were observed with IVIS.

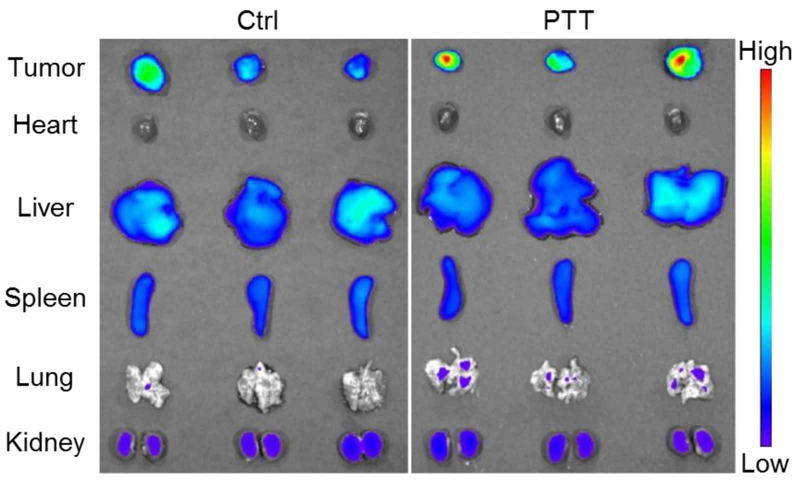

Supplementary Figure 23 More NPNs accumulated in PTT-treated tumors. DiD-labelled NPNs were *i.v.* injected into control or photothermally treated EMT6-bearing mice. After 48 h, the tumors and major organs of mice including heart, liver, spleen, lung, and kidney were collected and fluorescent images of these organs were acquired with IVIS. Data are representative of two independent experiments.

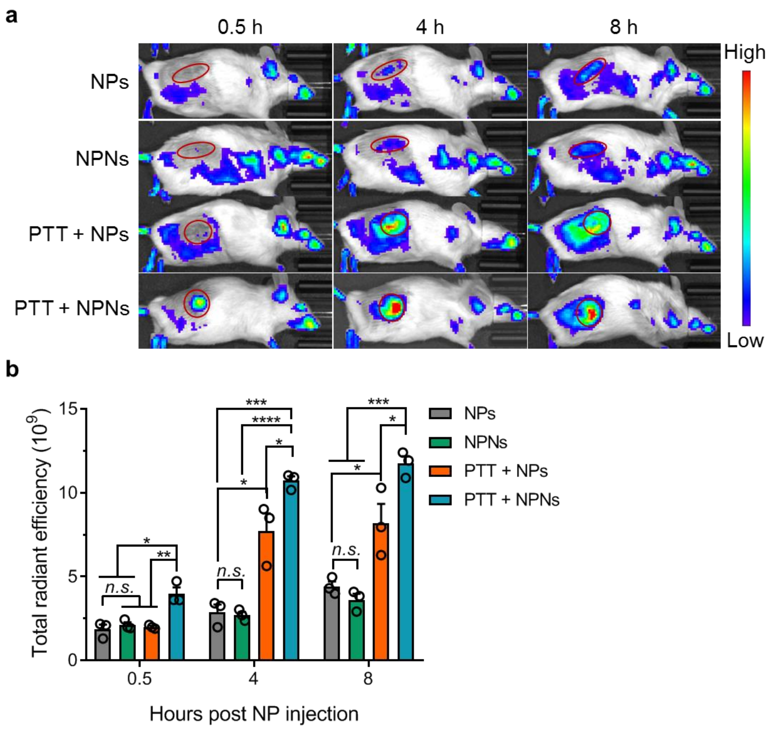

Supplementary Figure 24 PTT enhanced tumor accumulation of NPNs@DiD compared with PEG-*b*-PLGA NPs. DiD labelled NPs and NPNs were *i.v.* injected into PTT-treated or untreated EMT6-bearing mice. **a** At the indicated time points, *in vivo* DiD fluorescent signals were observed with IVIS. **b** Quantitative ROI analysis of DiD fluorescent signals in tumor areas. $n = 3$ per group. Data are shown as mean ± SEM and analyzed by unpaired two-tailed Student's *t*-test. *$P < 0.05$, **$P < 0.01$, ***$P < 0.001$, and ****$P < 0.0001$. *n.s.*, not significant. Source data are provided as a Source Data file.

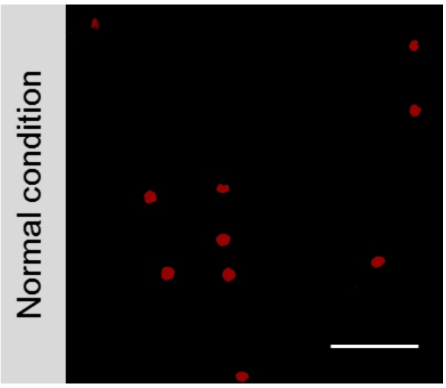

Supplementary Figure 25 Confocal fluorescent image of PI-stained neutrophils cultured under normal condition for 5 h. Scale bar, 50 μm. Data are representative of two independent experiments.

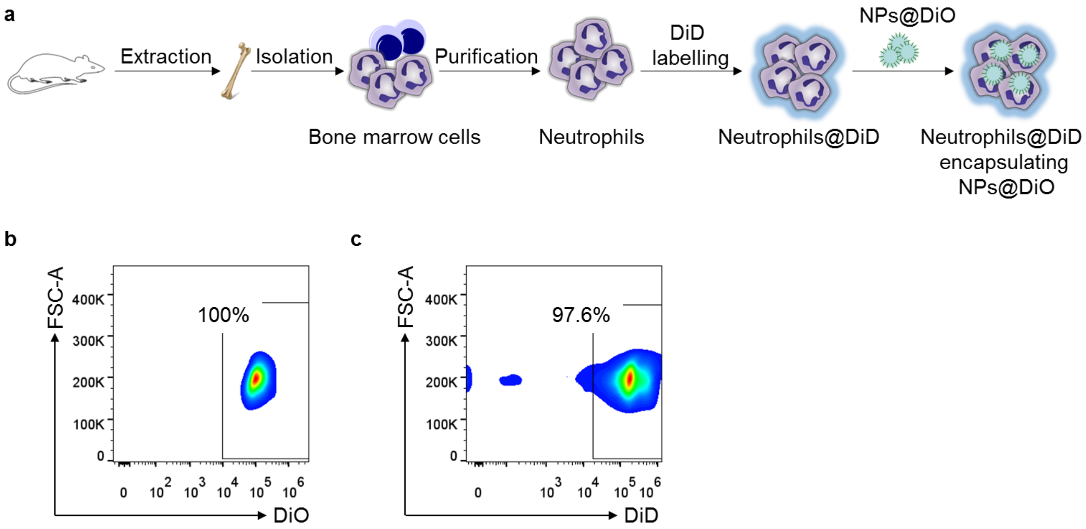

Supplementary Figure 26 Characteristics of transferred neutrophils. **a** Schematic showing the preparation of neutrophils@DiD encapsulating NPs@DiO. Neutrophils were isolated from bone marrow, labelled with DiD, and incubated with DiO-labelled PEG-*b*-PLGA NPs. The percentages of neutrophils (**b**) encapsulated with NPs@DiO and (**c**) labelled with DiD were analyzed by flow cytometry.

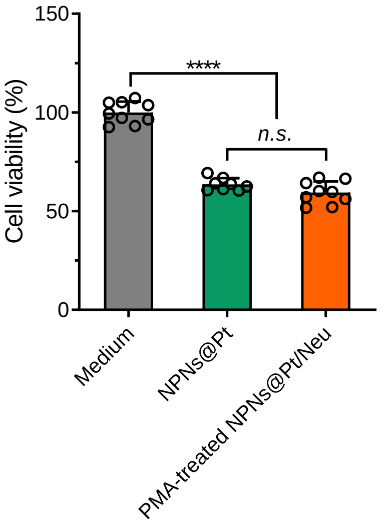

Supplementary Figure 27 Cytotoxicity of naïve NPNs@Pt and NPNs@Pt released from PMA-treated neutrophils against EMT6 cells. Neutrophils containing NPNs@Pt were treated with 100 nM PMA for 4 h. Then naïve NPNs@Pt or the supernatant containing NPNs@Pt released from neutrophils was added to EMT6 cells, at the cisplatin concentration of 15 μg mL^{-1}. After 24 h, the viability of EMT6 cells was detected using the MTT assay. n = 8 biologically independent samples for NPNs@Pt group and n = 9 biologically independent samples for the other two groups. Data are shown as mean ± SEM and analyzed by unpaired two-tailed Student's t-test. ****$P < 0.0001$. *n.s.*, not significant. Source data are provided as a Source Data file.

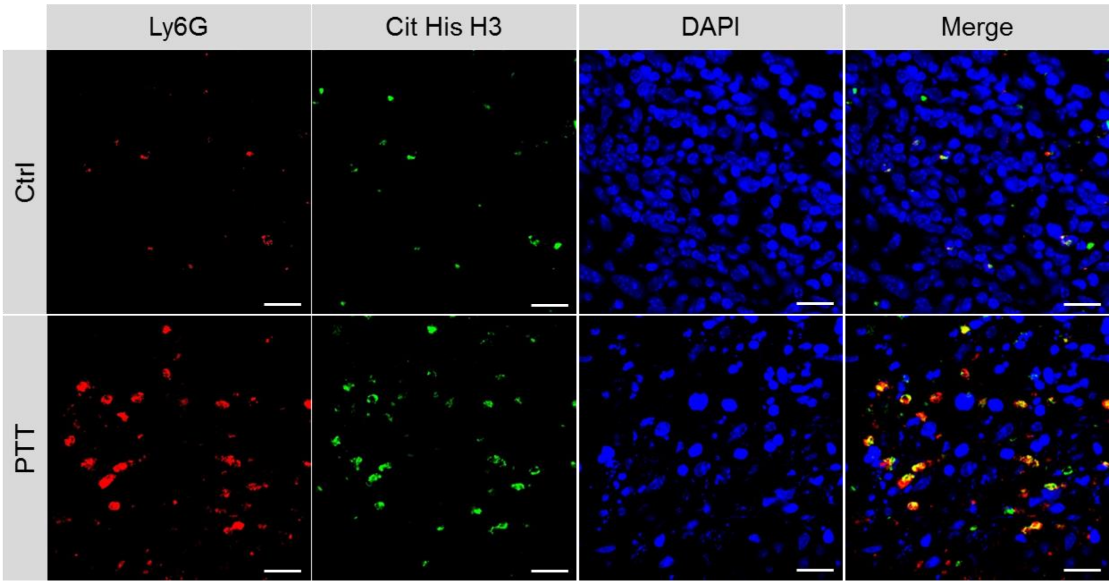

Supplementary Figure 28 *In vivo* NETosis in PTT-treated tumor. EMT6-bearing mice were left untreated or treated with PTT (40 ºC for 5 min). After 24 h, the tumors were excised and were stained with Ly6G antibody and antibody of citrullinated histone H3 (Cit-histone H3) to analyze *in vivo* NETosis after PTT treatment. Scale bars, 20 μm. Data are representative of three biological replicates.

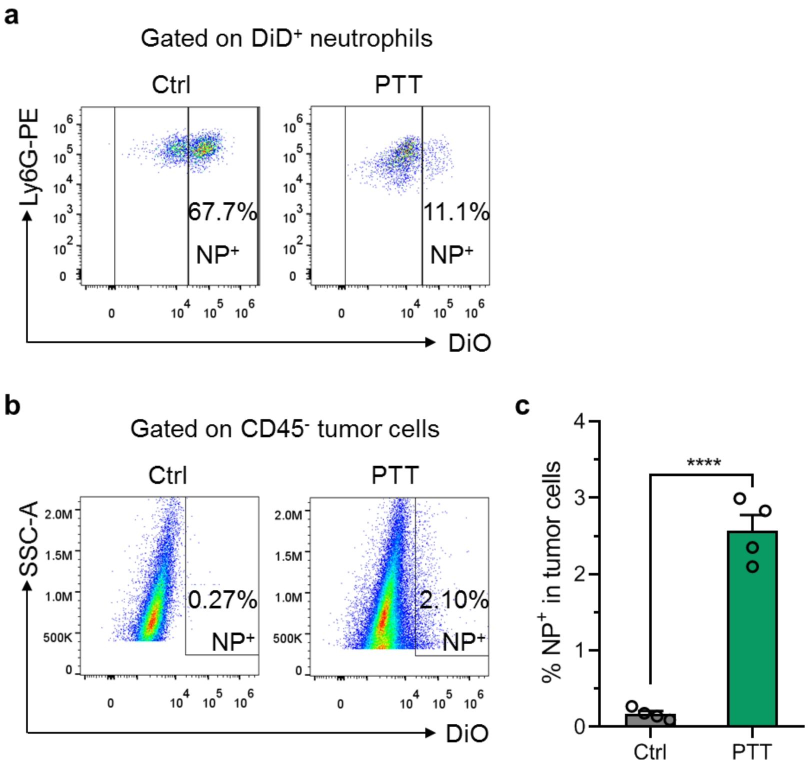

Supplementary Figure 29 Tumor cells internalized NPs that were released from neutrophils after the PTT treatment. EMT6-bearing mice were left untreated or were *i.v.* injected with NPs@PBT on -12 h and tumors were performed with PTT (40 ºC for 5 min) on -0.5 h. DiD-labelled neutrophils were incubated with DiO-labelled PEG-*b*-PLGA NPs (NPs@DiO) and intratumorally injected into the mice at 0 h. After 20 h, DiO fluorescent signals were analyzed by flow cytometry. **a** Flow cytometric measurements of percentage of DiD-labelled neutrophils that contained NPs@DiO. (**b**) Flow cytometric measurements and (**c**) corresponding quantitative analysis of percentage of tumor cells that internalized NPs@DiO released from transferred neutrophils. $n = 4$ per group. Data are shown as mean ± SEM and analyzed by unpaired two-tailed Student's *t*-test. ****$P < 0.0001$. Source data are provided as a Source Data file.

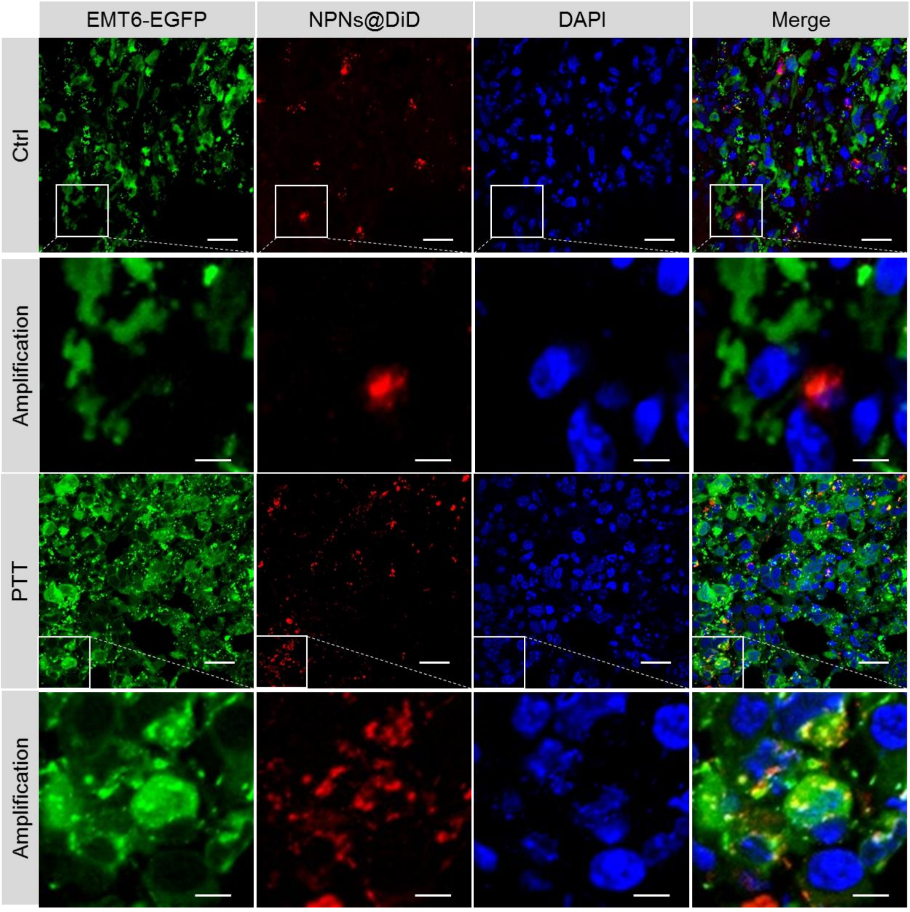

Supplementary Figure 30 EGFP expressing EMT6 tumor cells (EMT6-EGFP) internalized NPNs@DiD released by neutrophils in PTT-treated tumors. EMT6-EGFP bearing mice were left untreated or treated with PTT (40 ºC for 5 min). After 30 min, neutrophils encapsulated with DiD-labelled NPNs (NPNs@DiD) were intratumorally injected into the mice. After another 9 h, DiD fluorescent signals were analyzed by immunofluorescence. Scale bars, 20 μm for the amplification panels, and 100 μm for all other panels. Data are representative of three biological replicates.

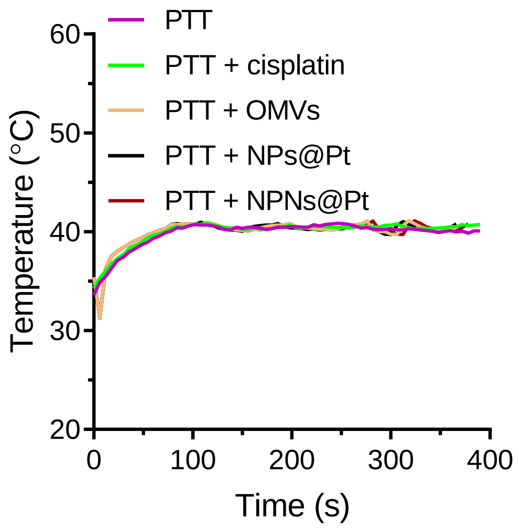

Supplementary Figure 31 Temperature change curves of tumor site during PTT treatment. Source data are provided as a Source Data file.

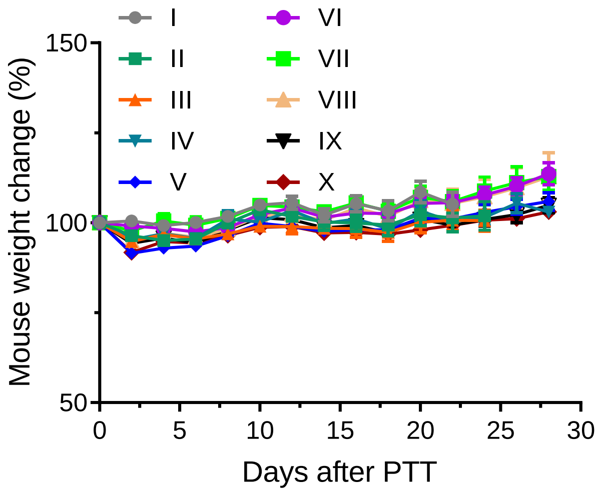

Supplementary Figure 32 Weight change curves of mice after different treatment. (I) PBS, (II) cisplatin, (III) OMVs, (IV) NPs@Pt, (V) NPNs@Pt, (VI) PTT, (VII) PTT + cisplatin, (VIII) PTT + OMVs, (IX) PTT + NPs@Pt, and (X) PTT + NPNs@Pt. $n = 5$ per group. Data are shown as mean ± SEM. Source data are provided as a Source Data file.

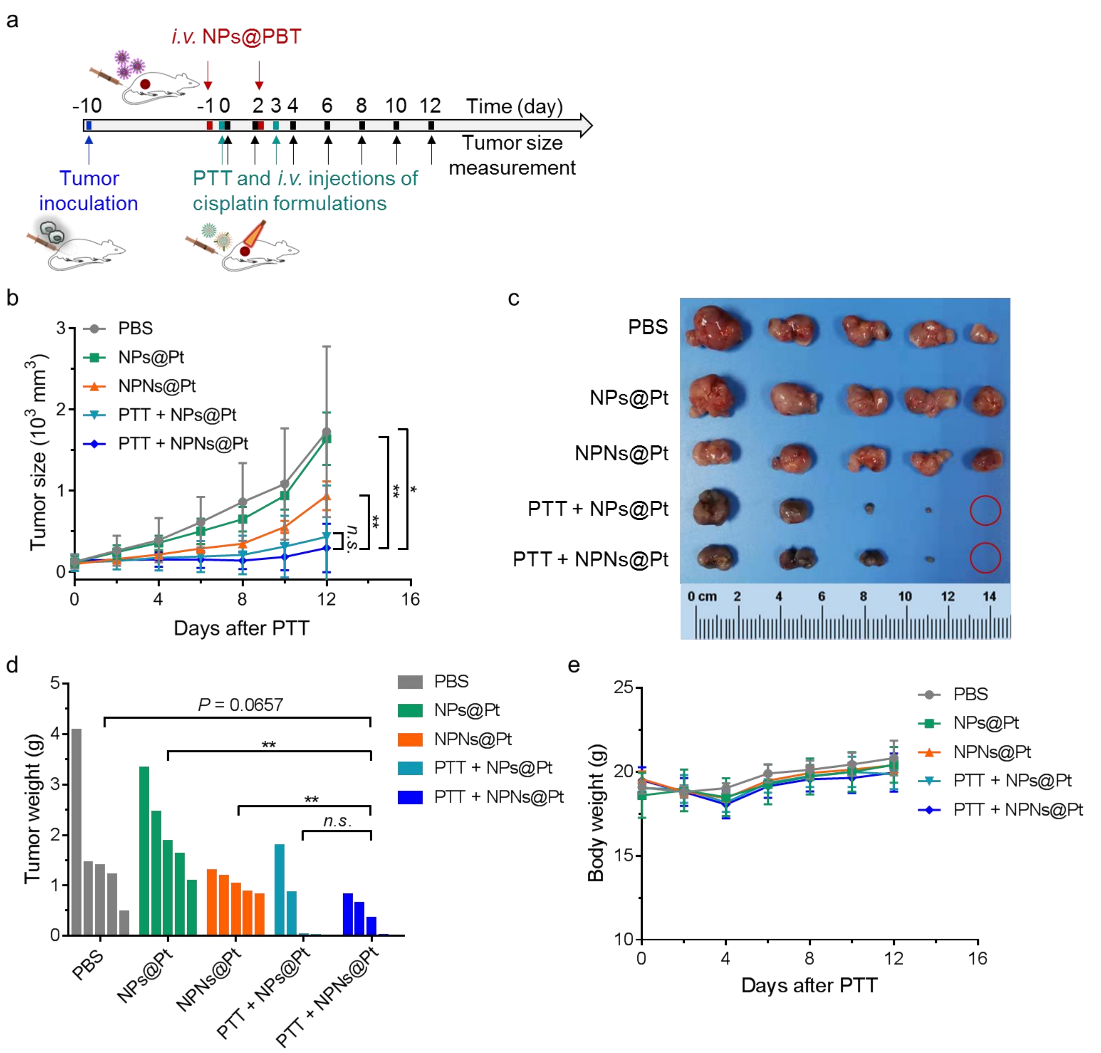

Supplementary Figure 33 Combined therapy of PTT with NPNs@Pt in CT26 tumor model. **a** Workflow for treatment of CT26-bearing mice using PTT-based neutrophil-mediated NPNs@Pt delivery system. On days 0 and 3 (defined as the 10th and 13th day after tumor inoculation), the mice received PTT treatment on the tumors followed by an *i.v.* injection of PBS or different formulations of cisplatin. Tumor volumes were measured every two days from day 0 to day 12. $n = 5$ per group. **b** Tumor growth curves during the treatments. (**c**) Photos and (**d**) weight of the tumors collected on day 13. **e** Change of mouse weight during the treatments. Data are shown as mean ± SEM and analyzed by unpaired two-tailed Student's *t*-test. $^*P < 0.05$, $^{**}P < 0.01$. *n.s.*, not significant. Source data are provided as a Source Data file.

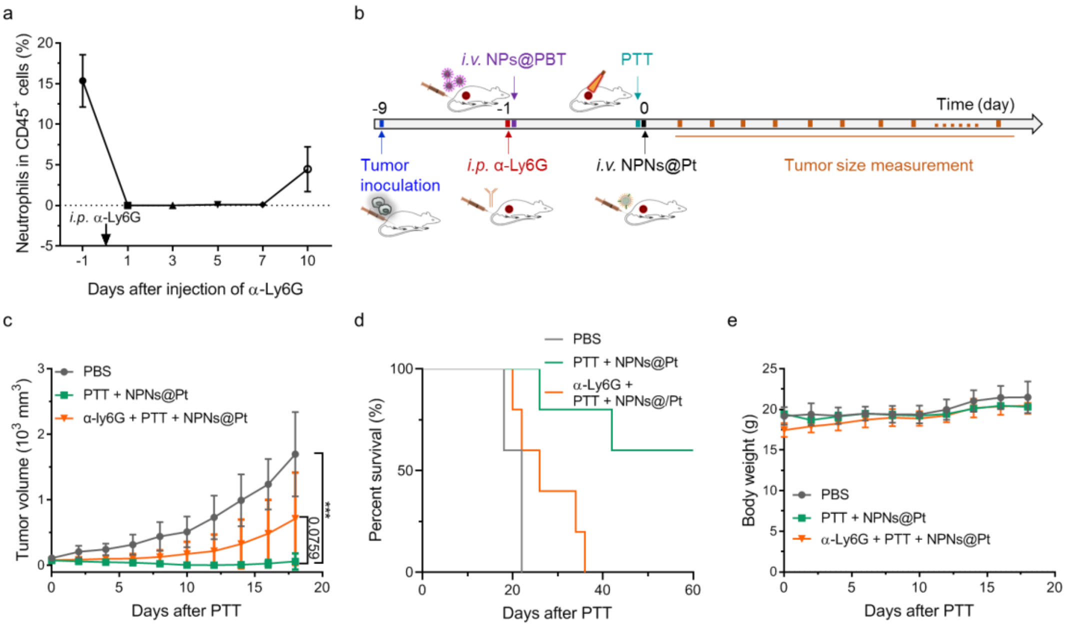

Supplementary Figure 34 Neutrophil depletion impaired the therapeutic efficacy of PTT plus NPNs@Pt. **a** The percentage of neutrophils in blood at indicated time points after receiving an *i.p.* injection of 200 μg α-Ly6G into mice. n = 3. **b** Workflow for determining the role of neutrophils in the therapeutic efficacy of PTT plus NPNs@Pt on EMT6-bearing mice. On day -1 (defined as the 8th day after tumor inoculation), the mice were treated with an *i.p.* injection of α-Ly6G followed by an *i.v.* injection of NPs@PBT. On day 0, the mice were received PTT treatment (40 °C, 5 min) on the tumors followed by *i.v.* injection of NPNs@Pt (2 mg cisplatin per kg body weight). Tumor volumes were measured every two days from day 0 to day 18. **(c-e)** Tumor growth curves (**c**), percent of survival (**d**), and mouse body weight (**e**) during the experiment. n = 5 per group. Data are shown as mean ± SEM and analyzed by unpaired two-tailed Student's t-test. ***P < 0.001. Source data are provided as a Source Data file.

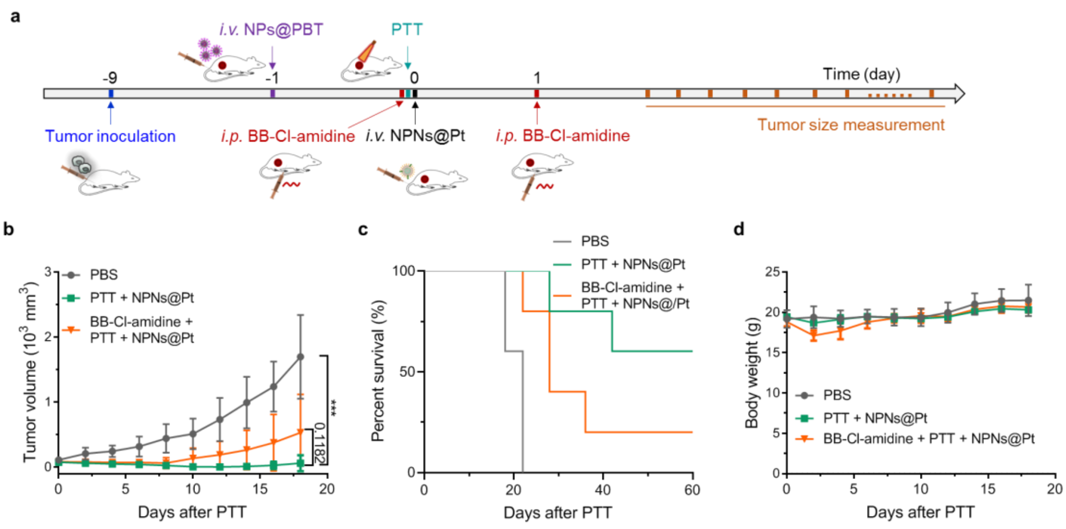

Supplementary Figure 35 Inhibiting NETosis with BB-Cl-amidine impaired the therapeutic efficacy of PTT with NPNs@Pt. **a** Workflow for determining the role of NETosis in the therapeutic efficacy of PTT plus NPNs@Pt on EMT6-bearing mice. On day -1 (defined as the 8th day after tumor inoculation), the mice were injected with NPs@PBT. On day 0, mice were *i.p.* injected with 160 µg BB-Cl-amidine to inhibit NETosis. After 30 min, the mice were received PTT treatment (40 °C, 5 min) on the tumors followed by *i.v.* injection of NPNs@Pt (2 mg cisplatin per kg body weight). On day 1, the mice were received an additional injection of 160 µg BB-Cl-amidine. Tumor volumes were measured every two days from day 0 to day 18. **(b-d)** Tumor growth curves (**b**), percent of survival (**c**), and mouse body weight (**d**) during the experiment. $n = 5$ per group. Data are shown as mean ± SEM and analyzed by unpaired two-tailed Student's t-test. ***$P < 0.001$. Source data are provided as a Source Data file.

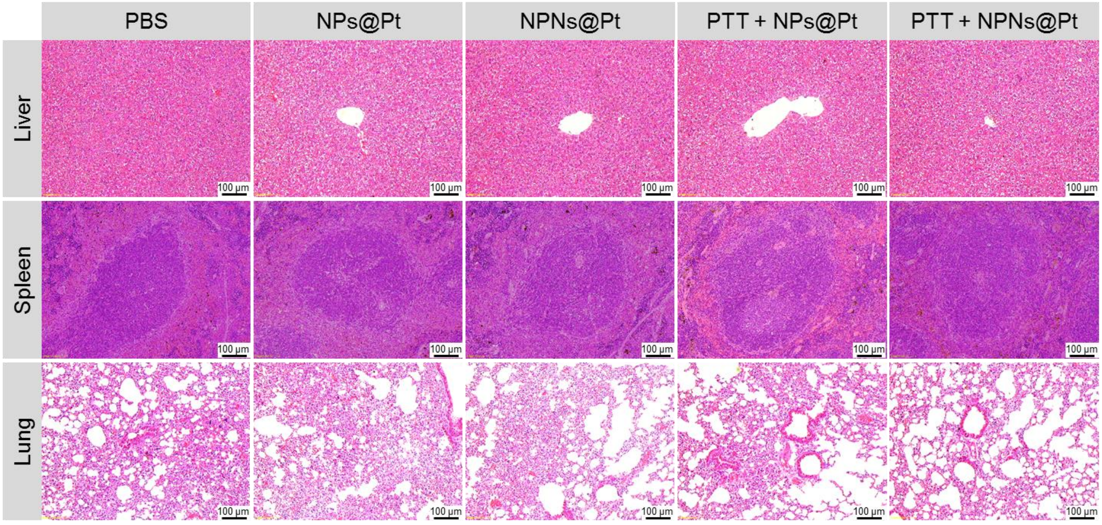

Supplementary Figure 36 Representative images of H&E staining of liver, spleen, and lung obtained at the end point of experiment. Scale bars, 100 μm. Data are representative of five biological replicates.
