# Supplementary material for: Chemotaxis-driven delivery of nano-pathogenoids for complete eradication of tumors post-phototherapy
Source: Nat Commun. 2020 Feb 28;11:1126. doi: 10.1038/s41467-020-14963-0 (PMC7048836; doi:10.1038/s41467-020-14963-0)
Supplement: Supplementary file 3 — Description of Supplementary files [file 41467_2020_14963_MOESM3_ESM.docx]

**Description of Additional Supplementary Files**

File Name: Supplementary Movie 1

**Supplementary Movie 1.** The track of neutrophils in/out of tumor blood vessels after PTT. Dorsal-skin-fold window chamber EMT6 tumor-bearing mice were anaesthetized by intraperitoneally injecting pentobarbital, photothermally treated with 40 ºC for 5 min, and *i.v.* injected with 2 μg PE-Ly6G antibody and NPs@DiO (3 μg DiO). Then the mice were put on a coverslip with tumor above the 20 × objective lens. After selecting a blood vessel in tumor, neutrophils in the blood vessel were photographed once every minute.

File Name: Source Data

**Source Data.** The Source Data file provides the raw data underlying Figures 2-8 and Supplementary Figures 1, 3, 7, 8, 10-12, 14, 17-19, 21, 24, 27, 29, 31-35.
