# Supplementary material for: Chemotaxis-driven delivery of nano-pathogenoids for complete eradication of tumors post-phototherapy
Source: Nat Commun. 2020 Feb 28;11:1126. doi: 10.1038/s41467-020-14963-0 (PMC7048836; doi:10.1038/s41467-020-14963-0)
Supplement: Supplementary file 5 — Reporting Summary [file 41467_2020_14963_MOESM5_ESM.pdf]

natureresearch

Corresponding author(s): Yucai Wang
Last updated by author(s): Feb 4, 2020

Reporting Summary

Nature Research wishes to improve the reproducibility of the work that we publish. This form provides structure for consistency and transparency in reporting. For further information on Nature Research policies, see Authors & Referees and the Editorial Policy Checklist .

Statistics

For all statistical analyses, confirm that the following items are present in the figure legend, table legend, main text, or Methods section.

n/a	Confirmed	
☐	☒	The exact sample size (*n*) for each experimental group/condition, given as a discrete number and unit of measurement
☐	☒	A statement on whether measurements were taken from distinct samples or whether the same sample was measured repeatedly
☐	☒	The statistical test(s) used AND whether they are one- or two-sided<br>*Only common tests should be described solely by name; describe more complex techniques in the Methods section.*
☒	☐	A description of all covariates tested
☒	☐	A description of any assumptions or corrections, such as tests of normality and adjustment for multiple comparisons
☐	☒	A full description of the statistical parameters including central tendency (e.g. means) or other basic estimates (e.g. regression coefficient) AND variation (e.g. standard deviation) or associated estimates of uncertainty (e.g. confidence intervals)
☒	☐	For null hypothesis testing, the test statistic (e.g. *F*, *t*, *r*) with confidence intervals, effect sizes, degrees of freedom and *P* value noted<br>*Give P values as exact values whenever suitable.*
☒	☐	For Bayesian analysis, information on the choice of priors and Markov chain Monte Carlo settings
☒	☐	For hierarchical and complex designs, identification of the appropriate level for tests and full reporting of outcomes
☒	☐	Estimates of effect sizes (e.g. Cohen's *d*, Pearson's *r*), indicating how they were calculated

Our web collection on statistics for biologists contains articles on many of the points above.

Software and code

Policy information about availability of computer code

Data collection	None
Data analysis	No codes were used in data analysis.

For manuscripts utilizing custom algorithms or software that are central to the research but not yet described in published literature, software must be made available to editors/reviewers. We strongly encourage code deposition in a community repository (e.g. GitHub). See the Nature Research guidelines for submitting code & software for further information.

Data

Policy information about availability of data

All manuscripts must include a data availability statement. This statement should provide the following information, where applicable:

- Accession codes, unique identifiers, or web links for publicly available datasets
- A list of figures that have associated raw data
- A description of any restrictions on data availability

Data available on request from the authors and raw data underlying figures are provided as a Source Data file.

Field-specific reporting

Please select the one below that is the best fit for your research. If you are not sure, read the appropriate sections before making your selection.

☒ Life sciences ☐ Behavioural & social sciences ☐ Ecological, evolutionary & environmental sciences

For a reference copy of the document with all sections, see nature.com/documents/nr-reporting-summary-flat.pdf

Life sciences study design

All studies must disclose on these points even when the disclosure is negative.

Sample size	Sample size was determined based on the minumum number of animals / replicates and was described in figure legend.
Data exclusions	No data was excluded from the calculations unless the samples varied sharply.
Replication	Number of repeats are indicated in the figure legends.
Randomization	Animals were randomly divided into different groups.
Blinding	Blinding was not employed but at least two investigators were involved in tumor measurement to ensure the accuracy of the data.

Reporting for specific materials, systems and methods

We require information from authors about some types of materials, experimental systems and methods used in many studies. Here, indicate whether each material, system or method listed is relevant to your study. If you are not sure if a list item applies to your research, read the appropriate section before selecting a response.

Materials & experimental systems

n/a	Involved in the study
☐	☒ Antibodies
☐	☒ Eukaryotic cell lines
☒	☐ Palaeontology
☐	☒ Animals and other organisms
☒	☐ Human research participants
☒	☐ Clinical data

Methods

n/a	Involved in the study
☒	☐ ChIP-seq
☐	☒ Flow cytometry
☒	☐ MRI-based neuroimaging

Antibodies

Antibodies used

APC anti-mouse/human CD11b Antibody, clone M1/70, catalog n.101212, Biolegend
APC/Cy7 anti-mouse/human CD11b Antibody, clone M1/70, catalog n.101226, Biolegend
FITC anti-mouse/human CD11b Antibody, clone M1/70, catalog n.101206, Biolegend
PE/Cy7 anti-mouse/human CD11b Antibody, clone M1/70, catalog n.101216, Biolegend
PerCP/Cyanine5.5 anti-mouse/human CD11b Antibody, clone M1/70, catalog n.101228, Biolegend
FITC anti-mouse Ly-6G Antibody, clone 1A8, catalog n.127606, Biolegend
PE anti-mouse Ly-6G Antibody, clone 1A8, catalog n.127608, Biolegend
PerCP/Cy5.5 anti-mouse Ly-6G Antibody, clone 1A8, catalog n.127616, Biolegend
PE anti-mouse CD45 Antibody, clone I3/2.3, catalog n.147712, Biolegend
PE/Cy7 anti-mouse CD45 Antibody, clone I3/2.3, catalog n.147704, Biolegend
APC anti-mouse CD45 Antibody, clone 30-F11, catalog n.103112, Biolegend
Brilliant Violet 510™ anti-mouse CD45 Antibody, clone 30-F11, catalog n.103138, Biolegend
APC/Cyanine7 anti-mouse CD45.2 Antibody, clone 104, catalog n.109824, Biolegend
FITC anti-mouse CD45.2 Antibody, clone 104, catalog n.109806, Biolegend
APC anti-mouse F4/80 Antibody, clone BM8, catalog n.123116, Biolegend
PE anti-mouse F4/80 Antibody, clone BM8, catalog n.123110, Biolegend
Brilliant Violet 510™ anti-mouse CD11c Antibody, clone N418, catalog n.117338, Biolegend
APC anti-mouse Ly-6G/Ly-6C (Gr-1) Antibody, clone RB6-8C5, catalog n.108412, Biolegend
Alexa Fluor® 647 anti-mouse CD31 Antibody, clone 390, catalog n.102416, Biolegend
PE anti-mouse Ly-6C Antibody, clone HK1.4, catalog n.128008, Biolegend
PE/Cy7 anti-mouse CD49b Antibody, clone HMα2, catalog n.103518, Biolegend
APC/Cyanine7 anti-mouse CD3 Antibody, clone 17A2, catalog n.100222, Biolegend
Brilliant Violet 510™ anti-mouse/human CD45R/B220 Antibody, clone RA3-6B2, catalog n.103248, Biolegend
PerCP/Cyanine5.5 anti-mouse/human CD44 Antibody, clone IM7, catalog n.103032, Biolegend
Anti- Ki-67 Antibody, catalog n.ab15580, abcam
InVivoMab anti-mouse Ly6G, clone 1A8, catalog n. BE0075-1-5MG, BioXcell
Purified anti-mouse Ly-6G Antibody, clone 1A8, catalog n.127601, Biolegend
Purified anti-mouse CD16/32 Antibody, clone 93, catalog n.101302, Biolegend
Rabbit polyclonal to Histone H3 (citrulline R2+R8+R17), ab5103, Abcam
Anti-mouse TLR2 antibody, clone T2.5, mab2-mtlr2, InvivoGen

	Goat-anti-rat IgG (H+L) HRP, abs20031, Absin<br>Goat-anti-rabbit IgG (H+L) HRP, BL003A, Biosharp<br>Goat-anti-rabbit IgG antibody, FITC conjugate, abs20004ss, Absin
Validation	All antibodies were used according to the manufacturers' instructions.

Eukaryotic cell lines

Policy information about cell lines

Cell line source(s)	All cell lines used in the study were from ATCC and EMT6-EGFP cells were constructed to express EGFP in EMT6 cell line.
Authentication	None of the cell lines were authenticated.
Mycoplasma contamination	All cell lines used in the study were negative for mycoplasma contamination.
Commonly misidentified lines<br>(See ICLAC register)	None of the cell lines were misidentified.

Animals and other organisms

Policy information about studies involving animals; ARRIVE guidelines recommended for reporting animal research

Laboratory animals	Female Balb/c mice, male C57BL/6 mice, and Tlr4-/- mice, 6 ~ 8 weeks.
Wild animals	The present study did not involve in wild animals.
Field-collected samples	The present study did not involve in samples collected from the field.
Ethics oversight	The mouse experiments were approved by the animal care regulations of University of Science and Technology of China.

Note that full information on the approval of the study protocol must also be provided in the manuscript.

Flow Cytometry

Plots

Confirm that:

- [x] The axis labels state the marker and fluorochrome used (e.g. CD4-FITC).
- [x] The axis scales are clearly visible. Include numbers along axes only for bottom left plot of group (a 'group' is an analysis of identical markers).
- [x] All plots are contour plots with outliers or pseudocolor plots.
- [x] A numerical value for number of cells or percentage (with statistics) is provided.

Methodology

Sample preparation	Detailed sample preparation for each experiment is described in the Methods Section of the manuscript.
Instrument	Instrument used for data collection is described in the Methods Section of the manuscript
Software	Software used for data analysis is described in the Methods Section of the manuscript
Cell population abundance	The cell population abundance was detected by flow cytometry and described in the manuscript.
Gating strategy	FSC-A/SSC-A gates of the total cell population were performed to exclude cell debris followed by gating DAPI- cells to exclude dead cells. Then the live cells were analyzed according to research purpose of each experiment. Further detailed gating strategy was offered in the manuscript.

- [x] Tick this box to confirm that a figure exemplifying the gating strategy is provided in the Supplementary Information.
